# The Influence of Layer Thickness on the Microstructure and Mechanical Properties of M300 Maraging Steel Additively Manufactured by LENS^®^ Technology

**DOI:** 10.3390/ma15020603

**Published:** 2022-01-14

**Authors:** Natalia Rońda, Krzysztof Grzelak, Marek Polański, Julita Dworecka-Wójcik

**Affiliations:** Military University of Technology, Kaliskiego 2 Str., 00-908 Warsaw, Poland; natalia.ronda@student.wat.edu.pl (N.R.); krzysztof.grzelak@wat.edu.pl (K.G.); julita.dworecka@wat.edu.pl (J.D.-W.)

**Keywords:** additive manufacturing, laser engineered net shaping, LENS, LMD, maraging steel, M300, microstructure, mechanical properties, XRD, retained austenite, hardness, layer thickness

## Abstract

This work investigates the effect of layer thickness on the microstructure and mechanical properties of M300 maraging steel produced by Laser Engineered Net Shaping (LENS^®^) technique. The microstructure was characterized using light microscopy (LM) and scanning electron microscopy (SEM). The mechanical properties were characterized by tensile tests and microhardness measurements. The porosity and mechanical properties were found to be highly dependent on the layer thickness. Increasing the layer thickness increased the porosity of the manufactured parts while degrading their mechanical properties. Moreover, etched samples revealed a fine cellular dendritic microstructure; decreasing the layer thickness caused the microstructure to become fine-grained. Tests showed that for samples manufactured with the chosen laser power, a layer thickness of more than 0.75 mm is too high to maintain the structural integrity of the deposited material.

## 1. Introduction

Additive manufacturing (AM) is currently the most intensively developing production technology due to the wide possibilities of designing elements for different branches of industry. AM is the process of producing three-dimensional parts, layer by layer based on a CAD model. The high-power laser or electron beam used during manufacturing allows for full melting of metallic powders [1,2,3].

Currently, there are many technologies of 3D printing of metal objects. AM is divided into two major techniques, i.e., Powder Bed Fusion (PBF) and Direct Energy Deposition (DED). In PBF-based technologies, thermal energy selectively fuses regions of the powder bed. The main representative processes of PBF are Selective Laser Sintering (SLS) and Selective Laser Melting (SLM). In DED-based technologies focused thermal energy is used to fuse materials by melting as they are being deposited. The main representative processes of DED are Laser Metal Deposition (LMD) and Laser Engineered Net Shaping (LENS^®^) [1,2].

One of the most important advantages of AM is the ability to control parameters so that the microstructure of the final product can be controlled, which is also combined with the ability of the control of mechanical properties. The main processing parameters of the LENS^®^ process are laser power, scan speed and powder feed rate. Each variable, however, has a large influence on the deposited material. Most likely, laser power has the greatest effect on the mechanical properties of parts made by LENS^®^. Grain size has been shown to grow by increasing laser power (or better laser density), consequently reducing hardness and other mechanical properties [4]. It was also observed that as the laser power increases, the porosity decreases [5,6,7].

In additive manufacturing, if the process parameters are not optimal, many defects are likely to occur. Depending on the materials and method, the processing window may be narrow or relatively large. One of the key advantages of DED-based methods is the so-called self-adaptation of the process [8]. This is rarely described in the literature, since its existence proves that the molten pool size and, thus, the laser power density is not constant during deposition (especially during the first 10+ layers). In short, self-adaptation is a process during which the layer thickness is stabilized despite dynamic changes in heat exchange, too intensive of a powder flow or too high of a laser power. In laboratory practice, this effect is used to ensure that the deposition is stable and is realized in a way that causes significant overbuilding at several initial layers. Later, along with the sample height, the meltpool grows (due to the conical nature of the laser beam), which results in a decrease in the laser power density. This method of conducting the measurement usually results in very good remelting during deposition, so the only defects that are found are the gas pores or cracks in the case of some materials.

Maraging steel is an ultrahigh strength steel with a very low carbon content (0.03 wt.% maximum) that can be hardened by an aging heat treatment occurring after the martensitic phase transformation. Maraging steels have high hardenability and high strength combined with high toughness. The reason for the high strength of maraging steel is that intermetallic precipitates can be formed during aging, such as Ni_3_(Mo, Ti) and Fe_2_Mo. The rare combination of high strength and toughness makes it well suited for safety-critical aircraft structures that require high strength and damage tolerance [9,10,11]. The microstructure of the maraging steel produced classically by casting followed by hot working may strongly depend on the quality, purity and size of the product. In the annealed state followed by air cooling, when its produced in the form of small diameter rod, the uniaxial evenly distributed grains (<50 µm) are dominating [12], composed of lathe martensite with almost always some retained austenite [13]. The bigger the diameter of the product, the broader the grain size distribution and the higher the grains size in the central axis of the delivered bar. The high price of the material causes it to be applied only in applications that truly require a combination of ultrahigh tensile and yield strength with high hardness and temperature resistance. The typical applications are: production tools (extrusion press rams, pistons, springs), aerospace and aircraft parts (anchor rails, arresting hooks, gimbal ring pivots, load cells) military parts (rocket motor cases, lightweight portable bridges) and other general application industrial parts (hydraulic hoses, cable sockets, tensile testing equipment) [13].

A literature review on maraging steels produced by additive manufacturing showed that most research is focused on PBF-based techniques, while there are few DED methods for manufacturing. Additively manufactured maraging steel has a unique microstructure in comparison with that of conventionally processed steel. There are several effects that causes this condition. First, the melting and solidification takes place in a very limited volume (in the range of microliters); second, the strong convection in the molten metal pool causes the proper mixing of the alloying elements; third, the cooling rates in the range of tens of Kelvins per second during the solidification suppress the significant segregation, but also participation. Finally, the very specific type of microstructure is obtained with so-called cells, which are, in fact, the cross section of the dendrite arms. The very important aspect of the process is that the microstructure is very consistent over the whole volume of the manufactured part, if the heat exchange conditions are kept more or less the same. Table 1 shows an overview of the published mechanical properties of maraging steel. The mechanical properties of AM-produced maraging steel are comparable to those of conventionally produced materials, but are not identical. From the above literature, it can be seen that ductility is drastically reduced with increasing strength after heat treatment, which is caused by precipitation of intermetallic particles [3,14]. Mechanical properties depend on the parameters of additive manufacturing and heat treatment, which in the case of this steel is basically unavoidable since the built material possesses only half of the tensile strength that is obtained after the aging process.

Based on this short comparison, it can be seen that the properties of additively manufactured maraging steel (M300) can reach the levels of classically produced maraging steel. In such cases, considering the relatively large cost of the input material and heat treatment, the overall cost of the parts fabricated by laser deposition may be one of the most crucial factors allowing or limiting its application. One of the most effective ways of lowering the cost of additive manufacturing is shortening the deposition time. This can be performed practically in several ways. One of the simplest ways is to increase the layer thickness of the deposit, which theoretically may not be easy, but in practice can be simply performed just by changing the powder feed rate proportionally to a change in the thickness, sometimes with only a tiny change in laser power. In such cases, the time required for the manufacturing of the part shortens proportionally to the layer thickness and lowers the “machine time”, lowering the overall cost. However, the limitation here is the geometry of the clad, which in extreme cases may cause a lack of fusion due to the presence of too much powder compared to available energy, or simply geometrical issues causing overlapping of the clads if their height to width ratio is too large. The aim of this study was to investigate the effect of the layer thickness on the porosity, microstructure, tensile properties and microhardness of LENS^®^-manufactured M300 maraging steel parts. The hatch distance and laser power were kept constant. We showed that in a relatively broad range of layer thicknesses, the properties of the material remain at the desired level; however, at some point, a drastic loss of mechanical properties is observed. Nevertheless, we obtained a shortening of the processing time by at least half, compared to the set of parameters normally suggested by the suppliers of laser deposition systems, proving that at least in static load applications, the mechanical properties of deposited materials are maintained.

## 2. Materials and Methods

The initial powder of M300 maraging steel was provided by Carpenter Technology Corporation. The nominal chemical composition obtained from the manufacturer was confirmed by dispersive X-ray spectroscopy (EDS) (Table 2). Small differences are observed; however, considering the precision of both methods, it is rather impossible to judge any deviation from the nominal composition.

**Figure 1:**
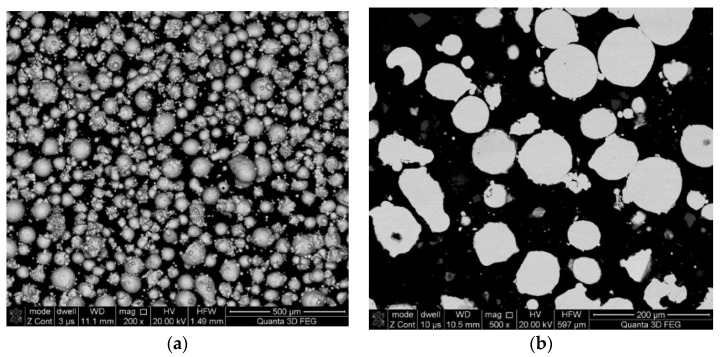
M300 maraging steel powder (**a**) morphology of the particles (SEM); (**b**) metallographic cross-section.

**Figure 2:**
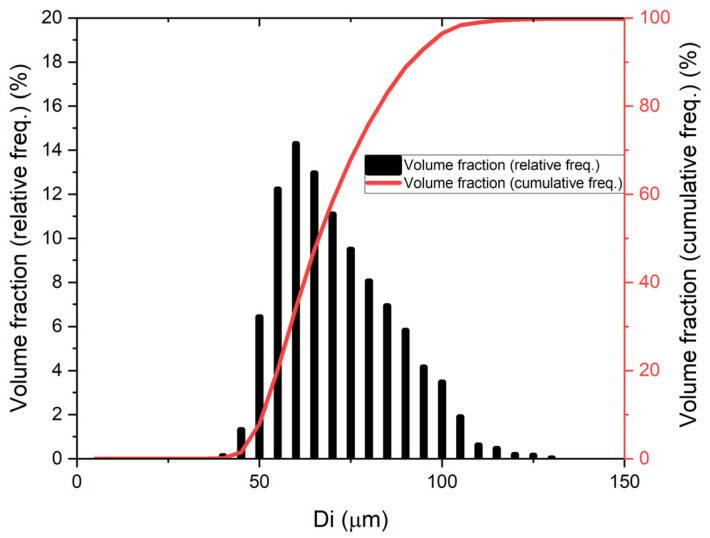
Particle size distribution of the M300 maraging steel powder used for the experiment.

The powder particles were observed using an SEM microscope (Quanta 3G FEM Dual Beam, FEI). As expected and declared by the manufacturer, particles were found to possess a spherical morphology, with a significant fraction of so-called satellites adhering to the surface of particles. The powder for the microstructural examination was mounted in thermoset resin, pressed in hot press for hot mounting of the metallographic specimens. In this way, powder particles were incorporated into the thermoset resin. Later on, the sample was ground on set of SiC sandpapers and polished with the use of proper diamond suspensions. Micrographs of the M300 powder are shown in Figure 1. The metallographic cross-section revealed a practical lack of internal porosity inside the particles. A particle size distribution analysis was performed by an IPS-U particle size meter (Kamika, Poland), and it confirmed that according to the specification, particle sizes were in the range of 40 to 130 μm (Figure 2), which was found to be the proper distribution for the chosen application. The measurement method of the IPS-U analyzer is complex and consists of measurement based on laser diffraction for the smallest particles and measurement of changes of scattered radiation beam for bigger particles. Combination of those methods allows eliminating the problems related to the purely diffraction-based measurement for larger particles. Ultrasonic powder feeder is used to provide the constant stream of the particles and improve the flowability.

Samples were subjected to X-ray diffraction (XRD) phase analysis using a Rigaku Ultima IV diffractometer (Co Kα radiation, λ = 1.78897 Å) with operating parameters of 40 mA and 40 kV in a 2θ range of 40–120°. Parallel beam geometry was used together with cross beam optics (CBO) to limit the influence of the surface roughness as well as Kβ radiation. Measurement was performed on non-rotating grinded samples. Quantitative analysis of retained austenite content was performed using PDXL (Rigaku, Tokyo, Japan) software using RIR method. The error of the analysis was estimated as not less than 5%, based on the users experience with standard samples. 

The test specimens were produced using a LENS 850-R system (Optomec) equipped with a 1 kW laser and atmosphere control system. Argon was used as the feed gas, and the process was made in a closed chamber, while the oxygen level was kept below 10 ppm during deposition.

**Figure 3:**
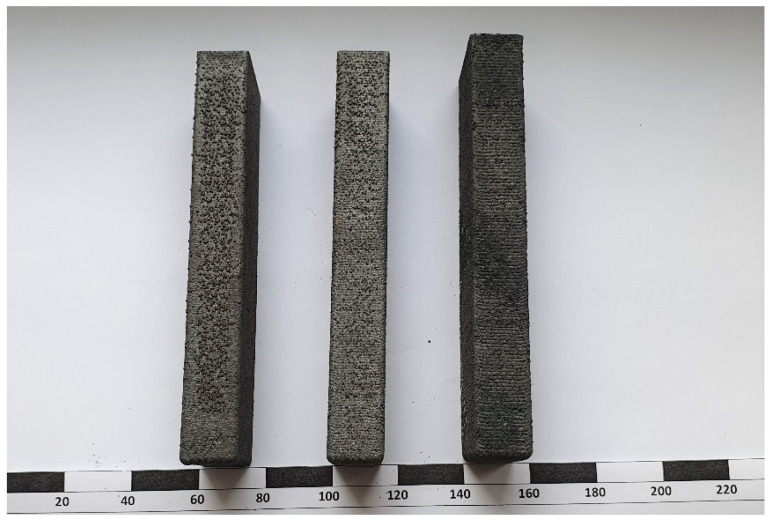
Photograph of the samples after heat treatment.

During the additive manufacturing process, the laser power was 600 W, and the layer thickness was changed from 0.5 to 1.0 mm with a hatch distance of 0.7 mm (Table 3). It is worth noting, however, that for that hatch value, “the usual” layer thickness would normally vary from as low as 0.15 to a maximum of 0.5 mm. In the preliminary studies, we found that a 0.25 mm layer thickness, or lower, resulted in very low porosities, and for that reason, we decided to use deposition parameters of much more challenging values. The laser travel speed was 12 mm/s for the contour and 15 mm for the inside part of the samples. Three rectangular specimens were produced with dimensions of 14.7 mm × 29.4 mm × 120 mm (XxYxZ). This allowed us to machine relatively large tensile specimens. Mild steel 20 mm thick substrate was used. No heating or cooling of the substrate was performed during the deposition. The as-deposited samples were cut from the substrate by electric discharge machining (EDM) and heat treated (840 °C 2 h + air cooling followed by 5 h aging at 480 °C and air cooling). Samples can be seen in Figure 3.

**Figure 4:**
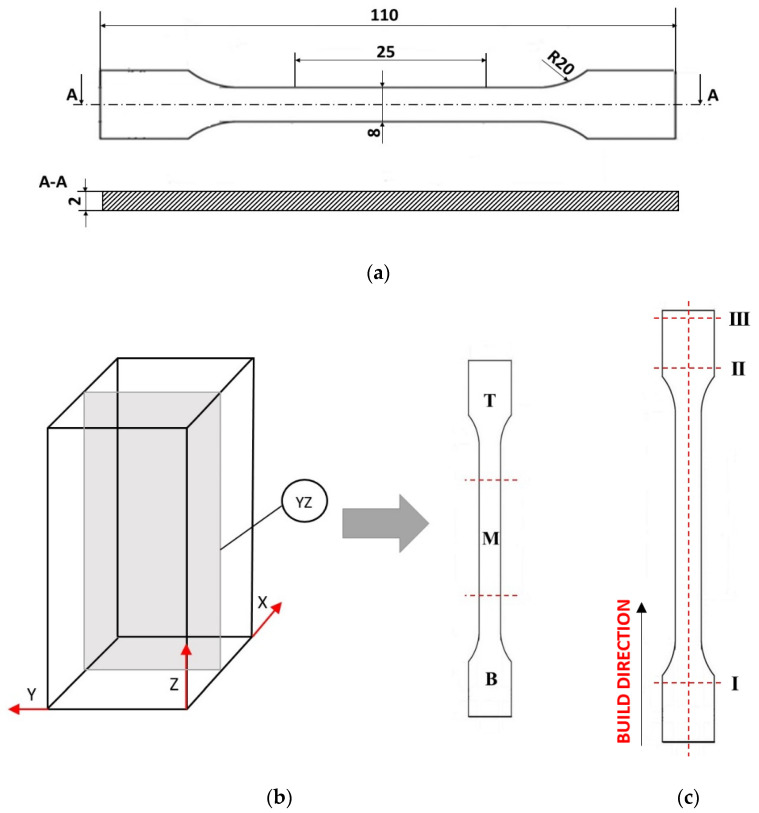
(**a**) Dimension of the tensile samples; (**b**) sample cutting diagram; (**c**) schematic of indentations performed for hardness testing.

Manufactured rectangular samples were cut into specimens for tensile testing (see Figure 4). Porosity and microstructure observations were performed on one of the cut slices but not on the tensile specimens (one specimen for each condition), which were then cut into three smaller pieces (B-bottom, M-middle, T-top) (Figure 4b). Porosity was assessed by light microscope images with the use of NIS-Elements software. The ratio of the total area of pores to the total selected area was calculated. Circularity was calculated also by software as: circularity = (4·Π·area)/m^2^.

Microstructural investigations were performed on a Keyence VHX-950F Digital Microscope and a scanning electron microscope on previously etched samples using aqua regia (HNO_3_:HCl = 1:3).

Vickers microhardness testing was carried out using an HMV-G Series Micro Vickers Hardness Tester under a load of 100 g (HV 0.1). The microhardness was measured along the entire length of the specimens through the center, and the indentations were 5 mm apart. In addition, seven indentations were made across each specimen approximately 28, 82 and 117 mm from the substrate, which are designated at points I, II, and III, respectively (Figure 4c).

The tensile tests were performed on an Instron 8501 testing machine. The force–elongation response of the material was recorded using a dynamic extensometer (Instron, 12.5 mm gauge length with a travel of ±5 mm). The size of the tensile sample is show in Figure 4a. Ten tensile samples were tested for every sample manufactured.

## 3. Results and Discussion

### 3.1. Porosity

Table 4 shows the calculated porosity for the various layer thicknesses divided into the bottom, middle and top of the sample. Three areas were chosen, since during the deposition process, the heat transfer conditions changed, which may have influenced the porosity. Table 5 shows the images of the porosity for the various layer thicknesses. A higher layer thickness resulted in a higher porosity. Moreover, the highest porosity was always at the bottom of the samples and the lowest was always at the top of the samples, which confirmed the mentioned assumption. The pores in the lower parts of the samples for each condition had the lowest circularity. Rounded pores were observed at the top of the samples. Spherical pores were the result of gas being trapped during melting of the metal, whereas irregular pores were formed due to incomplete melting between the fillets and/or layers resulting from inappropriate processing parameters [2,19,20].

### 3.2. Microstructure and Phase Composition

**Figure 5:**
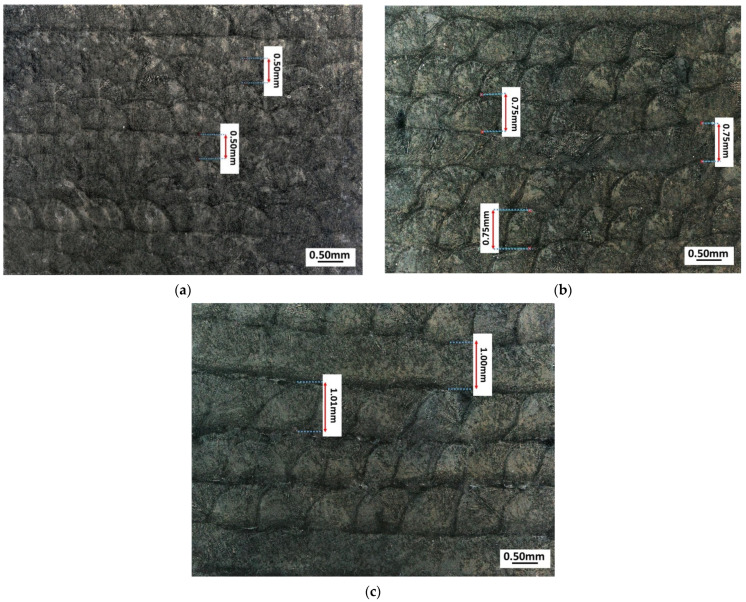
Microstructure of (**a**) sample A (layer thickness—0.5 mm); (**b**) sample B (layer thickness—0.75 mm); (**c**) sample C (layer thickness—1 mm).

Figure 5 shows low-magnification images of the microstructure of the obtained samples. Images show overlapping clad boundaries. In these studies, the melt pools were observed to range in depth from 0.5–1 mm, which resulted from the applied layer thickness. The melt pool boundaries caused by the 45° rotated scanning strategy can be clearly seen in the microstructure where laser scan tracks of every fourth layer run perpendicular.

**Figure 6:**
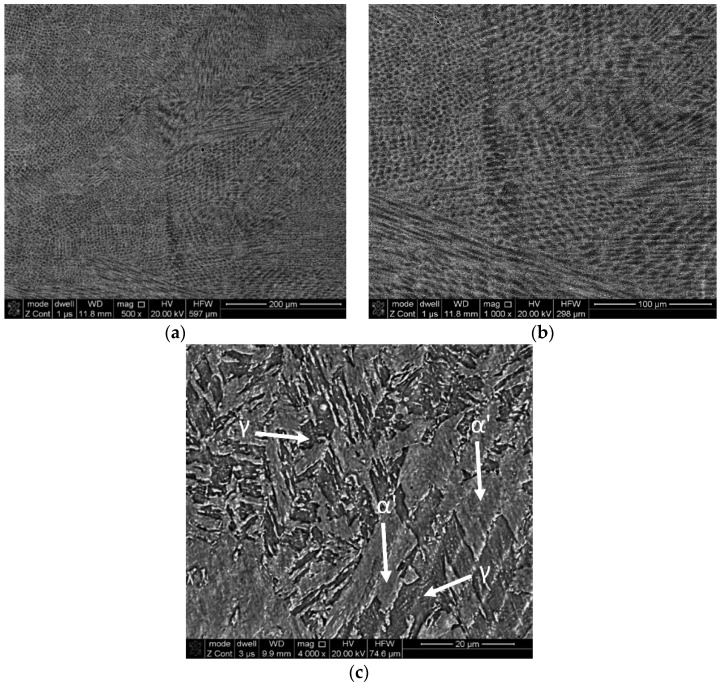
Scanning electron microscopy images of sample A (layer thickness—0.5 mm) at different magnifications ((**a**) 200 µm scale, (**b**) 100 µm scale, (**c**) 20 µm scale).

Examples of the high magnification micrographs taken by SEM are presented in Figure 6. Similar to other studies [15,16,17,18,21], a fine cellular dendritic structure was observed in the deposited material. These structures are typical microstructures of the LENS^®^ process due to supercooling together with a high velocity of solidification and dendritic growth [22]. Our observations show that a smaller layer thickness results in a finer microstructure, which is nothing extraordinary and can be explained by the fact that for smaller layer thicknesses, the material cools faster, causing the interdendritic arm spacing to be lower. Based on the metallographic observation, significant amounts of retained austenite were suspected to exist in the samples (Figure 6c) together with lath martensite. 

**Figure 7:**
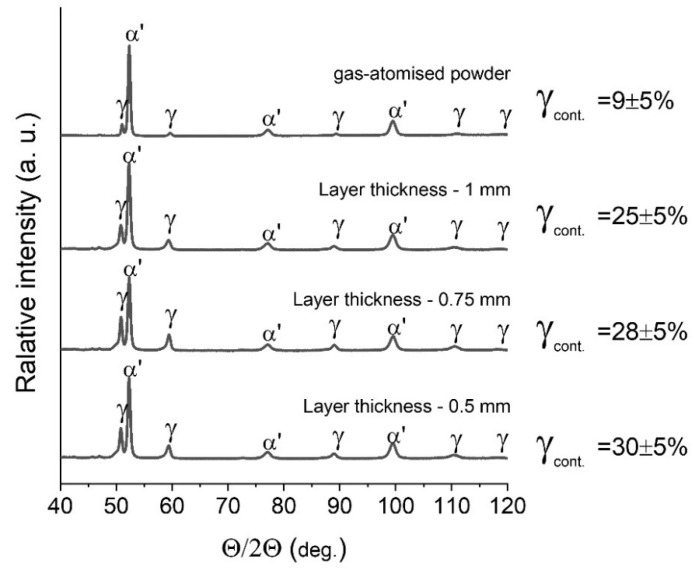
XRD patterns for samples manufactured with different parameters compared to the gas-atomized powder. Retained austenite content calculated with RIR method.

Following those observations, XRD phase analysis was performed to confirm the presence of retained austenite. Figure 7 shows the comparison of XRD patterns for manufactured and heat-treated samples in comparison to the initial powder of M300 steel. As can be seen, a significant amount of the retained austenite was found in each of the samples. It has to be underlined that, usually, the XRD-based quantitative analysis of the retained austenite is very difficult to perform and affected by many possible factors. Even the analysis made for the same sample but with a different method may result in obtaining the result that differs more than 10% of the absolute value. For that reason, the results should be treated more semi-quantitatively or even more qualitatively, and the differences in average values in the range of several percent should not be concluded as trends. For that reason, it cannot be confirmed here that any kind of corelation between the layer thickness and retained austenite content was observed in this case. The surprising observation, however, was the presence of the austenite in the gas atomized powder.

### 3.3. Microhardenss

**Figure 8:**
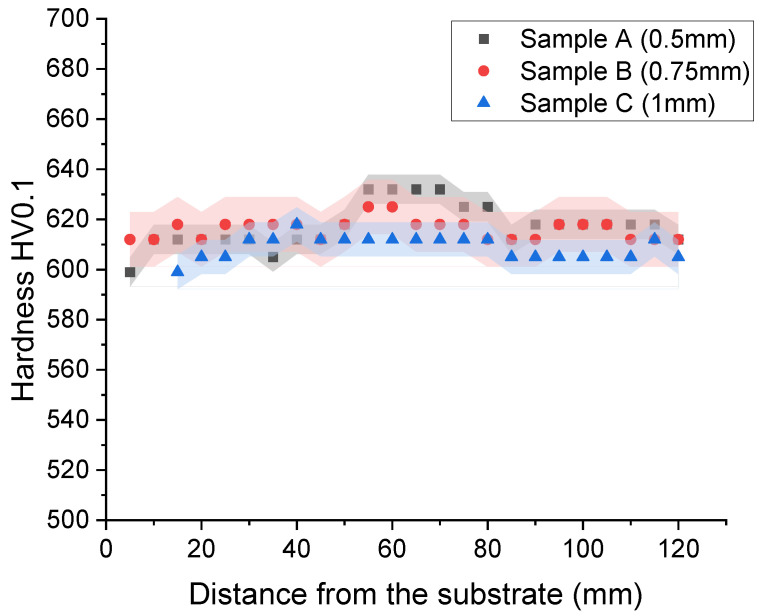
Graph of the microhardness distribution along samples.

An investigation of the microhardness of the samples was conducted. Twenty-four hardness indentations were performed along each sample. The hardness distribution along the samples (with 2 SD—standard deviations) is shown in Figure 8. The average value of hardness was found to be the highest, i.e., 617 HV0.1, in sample A (layer thickness—0.5 mm). Similar results, i.e., 616 HV0.1, were seen in sample B (layer thickness—0.75 mm). The lowest average value of hardness, i.e., 609 HV0.1, was found in sample C (layer thickness—1 mm) (Table 6). Considering the uncertainty of the results and phase analysis performed, we concluded that the observed differences are not really significant or may be related to the retained austenite content, however, it is definitely not easy to be proven. 

### 3.4. Tensile Properties

**Figure 9:**
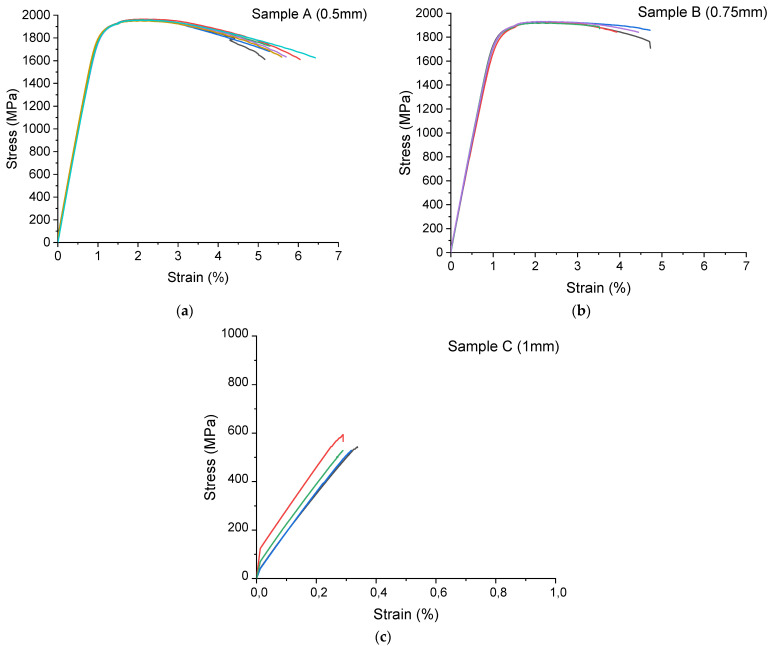
Tensile stress–strain curve for (**a**) sample A (layer thickness—0.5 mm); (**b**) sample B (layer thickness—0.75 mm); (**c**) sample C (layer thickness—1 mm). Overlapping curves on each graph represent the repetitions for the same sample state.

Figure 9 shows the tensile stress–strain curves for the maraging steel produced by LENS^®^ with different layer thicknesses, and Table 6 shows the average values of the strength properties with 2 standard deviations. For the sample with the lowest porosity (layer thickness—0.5 mm), the average value of tensile strength is 1958 ± 10 MPa, yield strength is 1856 ± 8 MPa, Young’s modulus is 194 ± 6 GPa and elongation is 4.8 ± 0.8%. For the sample with a layer thickness of 0.75 mm, the strength properties are slightly lower than those for the sample with a layer thickness of 0.5 mm. Parts with the highest porosity (layer thickness—1 mm) achieved the lowest properties—tensile strength is 548 ± 60 MPa, Young’s modulus is 178 ± 8 GPa and elongation is 0.19 ± 0.05%. Tensile tests showed that a reduction in the layer thickness leads to an increase in the tensile strength, yield strength, Young’s modulus and elongation. Most likely, the difference in the porosity of each sample was significant enough to result in very different tensile properties. From a practical point of view, however, the differences obtained for the 0.5 and 0.75 mm samples are rather insignificant. This result is quite surprising, since a height to hatch ratio of close to 1 is rather rarely used in laboratory practice and, therefore, little data can be found on the properties of such manufactured samples. It is worth noting that the manufacturing time of such a sample compared with a sample made with a “typical” layer thickness for such a beam geometry and laser power, which is normally close to 0.25 mm, is reduced three times, which may significantly lower the overall production cost. Despite having theoretically “some strength” (UTS~600 MPa) sample manufactured with 1 mm layer thickness is from an industrial application point of view totally useless since it basically loses cohesion and, by that, elongation is also at the critically low level.

**Figure 10:**
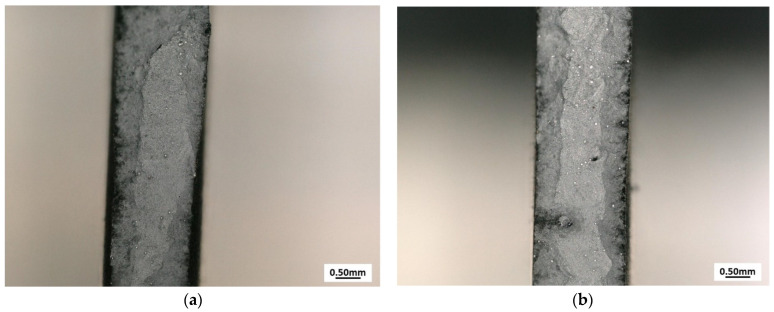

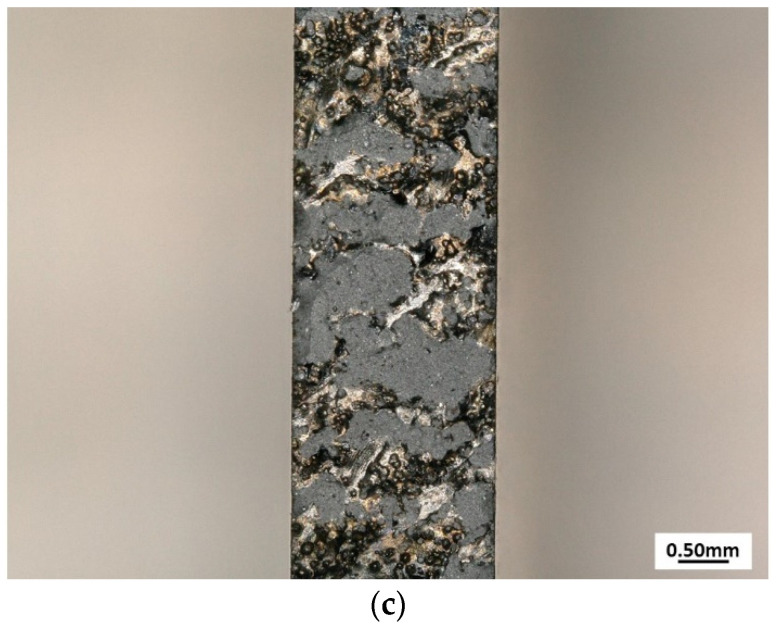
Fractures of (**a**) sample A (layer thickness—0.5 mm), (**b**) sample B (layer thickness—0.75 mm), (**c**) sample C (layer thickness—1 mm).

Fractures of samples after tensile testing were observed (Figure 10). The cracks for specimens with layer thicknesses of 0.5 mm and 0.75 mm occurred at an angle of 45° to the tensile direction, while the crack plane for the specimen with a layer thickness of 1 mm was perpendicular to the loading direction. At each fracture, unmelted powder particles and voids (porosity) were revealed. For samples A (layer thickness 0.5 mm) and B (layer thickness 0.75 mm), the unmelted particles and porosity were significantly lower than those for sample C (layer thickness 1 mm). Based on the tensile test results, specimens A and B were found to break after substantial plastic deformation. For specimen C, the fracture was found to be close to brittle (insignificant elongation at break), which shows that the layer thickness of the overlay of 1 mm is a threshold value, and parts made with this parameter or higher for 600 W laser power are not valuable for use in the industry. 

## 4. Conclusions

In this paper, a simple attempt to manufacture M300 maraging steel parts by the LENS^®^ method was made. The effect of layer thickness on the microstructure, metallurgical quality and mechanical properties of maraging steel was studied. Microscopic observation of the structure of the samples revealed a fine cellular structure. It was noted that a decrease in the layer thickness results in a decrease in the cell size and simultaneously lowers the porosity of the material. An increase in the layer thickness causes a small but observable deterioration of the material properties between the 0.5 and 0.75 mm layer thicknesses. Samples produced with a layer thickness of 1 mm were found to lose coherency during the tensile test and, for this reason, cannot be considered for use in any industrial application due to too low elongation at break. It was proven that for the chosen parameters and geometry, as compared to classically used layer thickness (0.25–0.5 mm), a significant increase in layer thickness can be successfully achieved, which shortens the manufacturing time and the overall cost. 

## Figures and Tables

**Table 1:** Overview of published mechanical properties of M300 maraging steel. AB: as built, SA: solution annealed, AH: aging heat treated.

Condition	Manufacturing Parameters	E [GPa]	UTS [MPa]	YS [MPa]	EL [%]	HRC
Wrought			1000–1170	760–895	6–15	35
Wrought AH [15]			1930–2050	1862–2000	5–7	52
SLM AB	Laser power: 285 W Scan speed: 950 mm/s Hatch space: 110 μm Layer thickness: 60 μm		1085 ± 19	920 ± 24	11.3 ± 0.3	35.7 ± 1.1
SLM AH 490/6 h			1942 ± 31	1867 ± 22	2.8 ± 0.1	52.9 ± 1.2
SLM SA 840/1 h			983 ± 13	923 ± 16	13.7 ± 0.7	27.5 ± 0.4
SLM SA AB840/1 h; 490/6 h [15]			1898 ± 33	1818 ± 27	4.8 ± 0.2	51.3 ± 0.9
SLM AB	Laser power: 105 W Scan speed: 200 mm/s Layer thickness: 30 μm	163 ± 4.4	1290 ± 112		13.3 ± 1.86	40
SLM AH 480/5 h [16]		189 ± 2.8	2217 ± 72		1.6 ± 0.26	58
SLM AB	Laser power: 100 W Scan speed: 150 mm/s Hatch space: 112 μm Layer thickness: 30 μm	163 ± 4.5	1290 ± 114	1214 ± 99		39.9 ± 0.1
SLM AH 480/5 h [17]		189 ± 2.9	2217 ± 73	1998 ± 32		58 ± 0.1
SLM AB	Laser power: 300 W Layer thickness: 50 μm		1214 ± 3	1135 ± 4	15 ± 2	35 ± 1
SLM SA AH 850/1 h; 490/6 h [18]			2106 ± 2	2055 ± 11	8 ± 2	53 ± 3

**Table 2:** Chemical composition of the M300 maraging steel.

M300 Maraging Steel Powder						
Element wt.%	Ni	Co	Mo	Ti	Si	Fe
Manufacturer	18.60	8.75	4.89	0.81	0.08	Bal.
EDS analysis	18.25 ± 0.13	9.15 ± 0.11	4.33 ± 0.05	0.86 ± 0.06	0.15 ± 0.04	Bal.

**Table 3:** Parameters of additive manufacturing process.

Sample	Power (W)	Hatch Space (mm)	Layer Thickness (mm)	Powder Feeding Rate (g/min)	Hatch Angle (◦)	Laser Head Travel Speed-Contour/Main (mm/s)	Laser Spot Size (mm)
A	600	0.7	0.50	9.3 ± 0.1	0/45/90/135	12/15	1.15
B	600	0.7	0.75	13.9 ± 0.1	0/45/90/135	12/15	1.15
C	600	0.7	1	19.8 ± 0.1	0/45/90/135	12/15	1.15

**Table 4:** Porosity measurement results. Results given with a single standard deviation. Circularity define shape of the pores and is calculated as: circularity = (4·Π·S)/L^2^, where: S—area of pore, L—length of the perimeter of the pore.

Layer Thickness		Porosity [%]	Circularity
0.5 mm	Bottom	0.21 ± 0.12	0.752 ± 0.003
	Middle	0.19 ± 0.08	0.780 ± 0.006
	Top	0.12 ± 0.07	0.785 ± 0.021
0.75 mm	Bottom	0.39 ± 0.13	0.718 ± 0.018
	Middle	0.26 ± 0.14	0.728 ± 0.016
	Top	0.21 ± 0.01	0.730 ± 0.010
1 mm	Bottom	1.12 ± 0.53	0.669 ± 0.027
	Middle	0.56 ± 0.20	0.767 ± 0.013
	Top	0.40 ± 0.13	0.780 ± 0.004

**Table 5:** Porosity of parts manufactured by LENS^®^.

Layer Thickness	Bottom	Middle	Top
0.5 mm	* 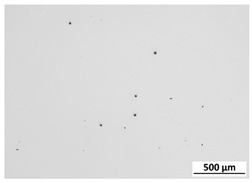 *	* 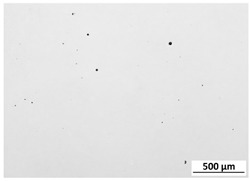 *	* 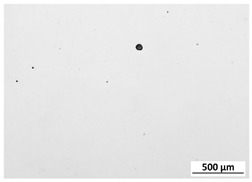 *
0.75 mm	* 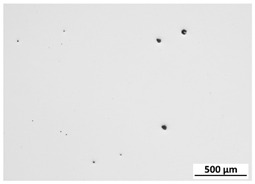 *	* 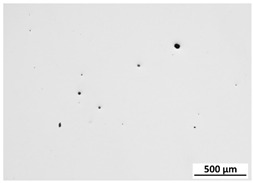 *	* 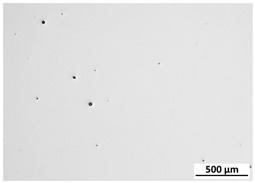 *
1 mm	* 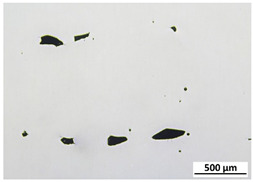 *	* 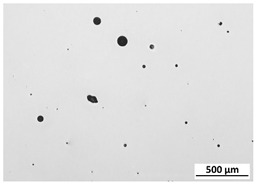 *	* 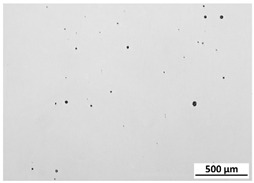 *

**Table 6:** Strength properties. Measurement uncertainty presented as 2 SD.

Layer Thickness	UTS [MPa]	YS [MPa]	E [GPa]	EL [%]	HV0.1
0.5 mm	1958 ± 10	1856 ± 8	194 ± 6	4.8 ± 0.8	617 ± 18
0.75 mm	1926 ± 10	1813 ± 20	188 ± 10	3.3 ± 1.0	616 ± 8
1 mm	548 ± 60	-	178 ± 8	0.19 ± 0.05	609 ± 8

## Data Availability

Raw data is available upon request.
